# Supplementary material for: Cell diversity and plasticity during atrioventricular heart valve EMTs
Source: Nat Commun. 2023 Sep 9;14:5567. doi: 10.1038/s41467-023-41279-6 (PMC10492828; doi:10.1038/s41467-023-41279-6)
Supplement: Supplementary file 1 — Supplementary Information [file 41467_2023_41279_MOESM1_ESM.pdf]

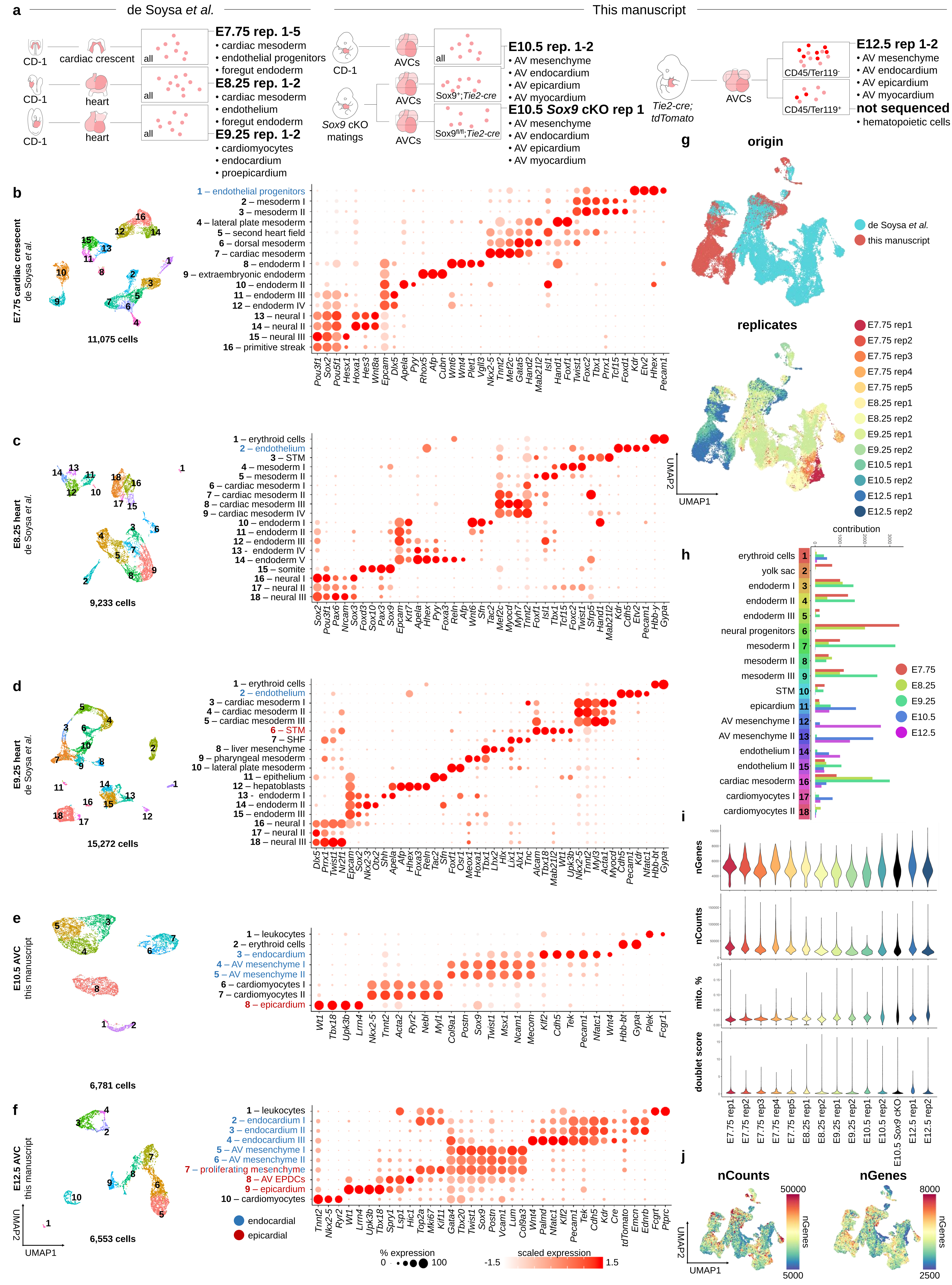

a
de Soysa et al.
This manuscript
CD-1
cardiac crescent
all
E7.75 rep. 1-5
• cardiac mesoderm
• endothelial progenitors
• foregut endoderm
heart
E8.25 rep. 1-2
• cardiac mesoderm
• endothelium
• foregut endoderm
E9.25 rep. 1-2
• cardiomyocytes
• endocardium
• proepicardium
AVCs
Sox9 cKO matings
Sox9+;Tie2-cre
Sox9fl/fl;Tie2-cre
E10.5 rep. 1-2
• AV mesenchyme
• AV endocardium
• AV epicardium
• AV myocardium
E10.5 Sox9 cKO rep 1
• AV mesenchyme
• AV endocardium
• AV epicardium
• AV myocardium
Tie2-cre; tdTomato
CD45/Ter119-
CD45/Ter119+
E12.5 rep 1-2
• AV mesenchyme
• AV endocardium
• AV epicardium
• AV myocardium
not sequenced
• hematopoietic cells
b
E7.75 cardiac cresecent de Soysa et al.
11,075 cells
1 – endothelial progenitors
2 – mesoderm I
3 – mesoderm II
4 – lateral plate mesoderm
5 – second heart field
6 – dorsal mesoderm
7 – cardiac mesoderm
8 – endoderm I
9 – extraembryonic endoderm
10 – endoderm II
11 – endoderm III
12 – endoderm IV
13 – neural I
14 – neural II
15 – neural III
16 – primitive streak
Pou3f1 Sox2 Pou5f1 Hesx1 Hoxa1 Hes3 Wnt8a Epcam Dlx5 Apela Pyy Rhox5 Afp Cubn Wnt6 Wnt4 Plet1 Vgll3 Nkx2-5 Tnnt2 Mef2c Gata5 Hand2 Mab21l2 Isl1 Hand1 Foxf1 Twist1 Foxc2 Tbx1 Prrx1 Tcf15 Foxd1 Kdr Etv2 Hhex Pecam1
c
E8.25 heart de Soysa et al.
9,233 cells
1 – erythroid cells
2 – endothelium
3 – STM
4 – mesoderm I
5 – mesoderm II
6 – cardiac mesoderm I
7 – cardiac mesoderm II
8 – cardiac mesoderm III
9 – cardiac mesoderm IV
10 – endoderm I
11 – endoderm II
12 – endoderm III
13 - endoderm IV
14 – endoderm V
15 – somite
16 – neural I
17 – neural II
18 – neural III
Sox2 Pou3f1 Pax6 Nrcam Sox3 Foxd3 Sox10 Pax3 Sox9 Epcam Krt7 Apela Hhex Pyy Foxa3 Reln Afp Wnt6 Sfn Tac2 Mef2c Myocd Myh7 Tnnt2 Foxf1 Isl1 Tbx1 Tcf15 Foxc2 Twist1 Sfrp5 Hand1 Mab21l2 Kdr Cdh5 Etv2 Pecam1 Hbb-y Gypa
d
E9.25 heart de Soysa et al.
15,272 cells
1 – erythroid cells
2 – endothelium
3 – cardiac mesoderm I
4 – cardiac mesoderm II
5 – cardiac mesoderm III
6 – STM
7 – SHF
8 – liver mesenchyme
9 – pharyngeal mesoderm
10 – lateral plate mesoderm
11 – epithelium
12 – hepatoblasts
13 - endoderm I
14 – endoderm II
15 – endoderm III
16 – neural I
17 – neural II
18 – neural III
Dlx5 Prrx1 Twist1 Nr2f1 Epcam Sox2 Nkx2-3 Otx2 Shh Apela Afp Hhex Foxa3 Reln Tac2 Sfn Foxf1 Osr1 Meox1 Hoxa1 Tbx1 Lhx2 Hlx Lix1 Alx1 Tnc Alcam Tbx18 Mab21l2 Wt1 Upk3b Nkx2-5 Tnnt2 Myl3 Acta1 Myocd Cdh5 Pecam1 Kdr Nfatc1 Hbb-bt Gypa
e
E10.5 AVC this manuscript
6,781 cells
1 – leukocytes
2 – erythroid cells
3 – endocardium
4 – AV mesenchyme I
5 – AV mesenchyme II
6 – cardiomyocytes I
7 – cardiomyocytes II
8 – epicardium
Wt1 Tbx18 Upk3b Lrrn4 Nkx2-5 Tnnt2 Acta2 Ryr2 Nebl Myl1 Col9a1 Postn Sox9 Twist1 Msx1 Ncam1 Mecom Klf2 Cdh5 Tek Pecam1 Nfatc1 Wnt4 Hbb-bt Gypa Plek Fcgr1
f
E12.5 AVC this manuscript
6,553 cells
UMAP2
UMAP1
1 – leukocytes
2 – endocardium I
3 – endocardium II
4 – endocardium III
5 – AV mesenchyme I
6 – AV mesenchyme II
7 – proliferating mesenchyme
8 – AV EPDCs
9 – epicardium
10 – cardiomyocytes
endocardial
epicardial
Tnnt2 Nkx2-5 Ryr2 Wt1 Lrrn4 Upk3b Tbx18 Spry1 Lsp1 Hic1 Top2a Mki67 Kif11 Gata4 Tbx20 Twist1 Sox9 Postn Vcam1 Lum Col9a3 Wnt4 Palmd Nfatc1 Klf2 Pecam1 Tek Cdh5 Kdr Cre tdTomato Emcn Ednrb Fcgrt Ptprc
% expression
0 100
scaled expression
-1.5 1.5
g
origin
de Soysa et al.
this manuscript
replicates
E7.75 rep1
E7.75 rep2
E7.75 rep3
E7.75 rep4
E7.75 rep5
E8.25 rep1
E8.25 rep2
E9.25 rep1
E9.25 rep2
E10.5 rep1
E10.5 rep2
E12.5 rep1
E12.5 rep2
UMAP2
UMAP1
h
contribution
erythroid cells 1
yolk sac 2
endoderm I 3
endoderm II 4
endoderm III 5
neural progenitors 6
mesoderm I 7
mesoderm II 8
mesoderm III 9
STM 10
epicardium 11
AV mesenchyme I 12
AV mesenchyme II 13
endothelium I 14
endothelium II 15
cardiac mesoderm 16
cardiomyocytes I 17
cardiomyocytes II 18
E7.75
E8.25
E9.25
E10.5
E12.5
i
nGenes
nCounts
mito. %
doublet score
E7.75 rep1
E7.75 rep2
E7.75 rep3
E7.75 rep4
E7.75 rep5
E8.25 rep1
E8.25 rep2
E9.25 rep1
E9.25 rep2
E10.5 rep1
E10.5 rep2
E10.5 Sox9 cKO
E12.5 rep1
E12.5 rep2
j
nCounts
nGenes
50000
5000
8000
2500
UMAP2
UMAP1

Supplementary Figure 1. Integration of single-cell datasets. Related to Fig. 1.

(a) Schematic outlining collection of single-cells for sequencing and target cell types. E7.75-E9.25 whole heart libraries were integrated from De Soysa *et al.* (2019), while E10.5 and E12.5 atrioventricular canal (AVC), including the *Sox9* conditional knockout (cKO) libraries are newly generated data. Red, *Tie2-cre*;tdTomato+ cells. (b-f), UMAPs displaying clusters and dot plots displaying select genes enriched within populations identified at (b) E7.75, (c) E8.25, (d) E9.25, (e) E10.5, and (f) E12.5. Size of the dot represents proportion of the cluster that expresses each gene. Colour indicates level of expression. Cluster annotations are coloured based on their inclusion in endothelial-derived and epicardial subsets. (g) UMAPs showing origin and replicate distribution in the complete dataset. (h) Timepoint contribution to each cluster identified in complete dataset. The frequency of endothelial and mesenchymal populations is increased in libraries generated at E10.5 and E12.5 due to the microdissection of AVCs at these stages. (i) Violin plots displaying quality metrics for complete dataset, displaying doublet score, mitochondrial percent, nCounts, and nGenes for each replicate. (j) UMAPs showing nCounts and nGenes distribution in complete dataset. AV, atrioventricular; AVC, atrioventricular canal; mito, mitochondrial; SHF, second heart field, STM, septum transversum mesenchyme. Source Data are provided as a Source Data file.

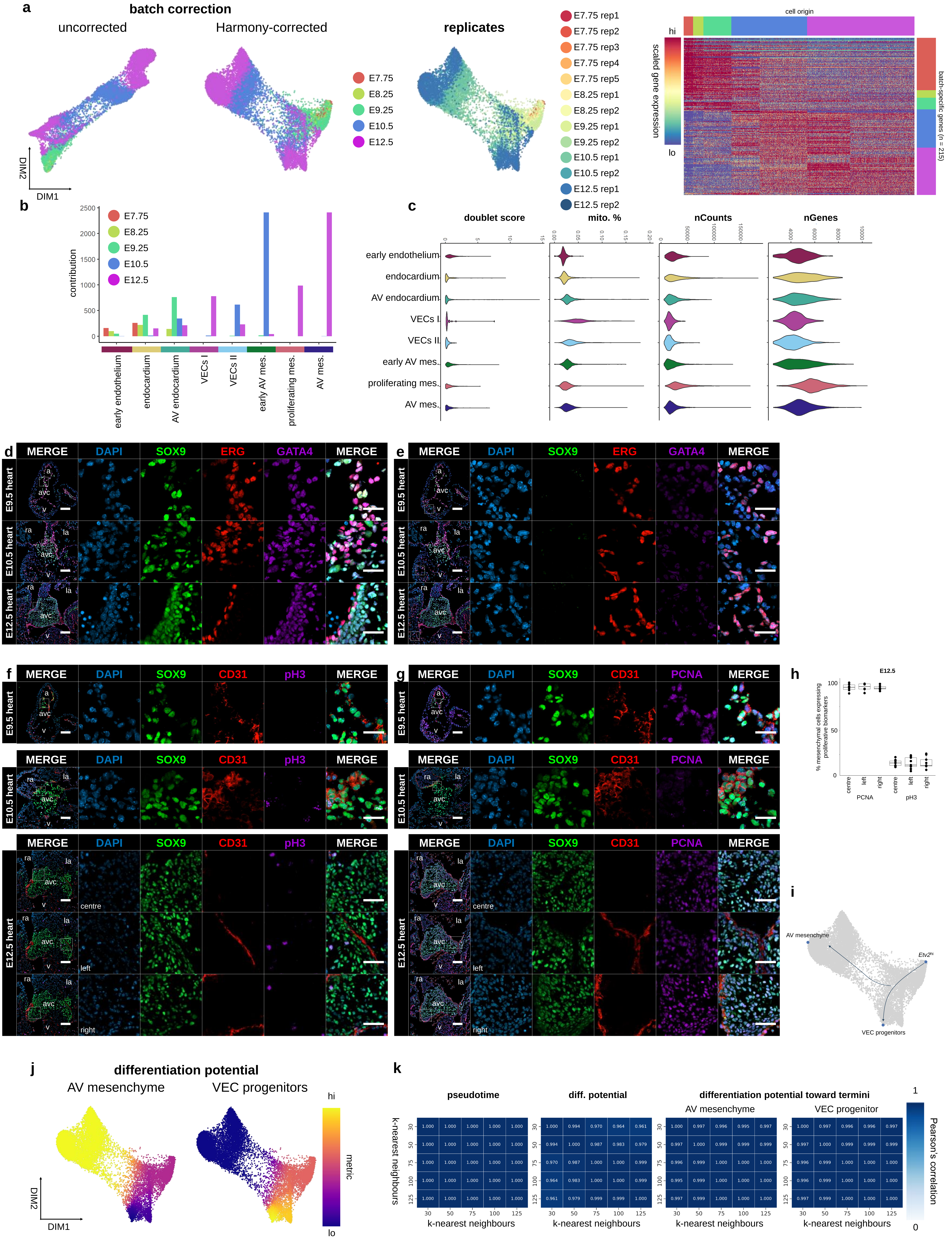
a
batch correction
uncorrected
Harmony-corrected
E7.75
E8.25
E9.25
E10.5
E12.5
DIM2
DIM1
replicates
E7.75 rep1
E7.75 rep2
E7.75 rep3
E7.75 rep4
E7.75 rep5
E8.25 rep1
E8.25 rep2
E9.25 rep1
E9.25 rep2
E10.5 rep1
E10.5 rep2
E12.5 rep1
E12.5 rep2
cell origin
hi
lo
scaled gene expression
batch-specific genes (n = 215)
b
contribution
early endothelium
endocardium
AV endocardium
VECs I
VECs II
early AV mes.
proliferating mes.
AV mes.
c
doublet score
mito. %
nCounts
nGenes
d
e
MERGE
DAPI
SOX9
ERG
GATA4
E9.5 heart
E10.5 heart
E12.5 heart
a
avc
v
ra
la
f
g
CD31
pH3
PCNA
centre
left
right
h
E12.5
% mesenchymal cells expressing proliferative biomarkers
PCNA
pH3
i
AV mesenchyme
Etv2hi
VEC progenitors
j
differentiation potential
AV mesenchyme
VEC progenitors
hi
metric
lo
k
pseudotime
diff. potential
differentiation potential toward termini
AV mesenchyme
VEC progenitor
k-nearest neighbours
Pearson's correlation

Supplementary Figure 2. Characterization of endocardial-derived lineages of the atrioventricular canal. Related to Fig. 2.

(a) Force-directed layouts and a heatmap showing timepoint distribution before and after Harmony correction as well as replicate distribution and batch-associated genes in endothelial-derived lineages. (b) Timepoint contribution to each cluster identified in endothelial-derived lineages. (c) Violin plots displaying doublet score, mitochondrial percent, nCounts, and nGenes for each cluster identified in endothelial-derived lineages. SOX9, ERG, and GATA4 staining in (d) AVCs and (e) ventricles at E9.25, E10.5, and E12.5, showing significantly higher expression of GATA4 within endocardial lineages of the AVC. Immunostaining of proliferative markers, including (f) phospho-histone H3 (pH3) and (g) PCNA, along with markers of mesenchymal (SOX9) and endothelial (CD31) identity in AVCs from E9.5, E10.5, and E12.5. (h) Quantification of proliferation across domains of the AVC at E12.5, showing no significant differences between central, left lateral, or right lateral cushions (two-tailed unpaired t-test). (i) Force-directed layout showing termini and rough trajectory for Palantir analysis. (j) Force-directed layouts showing differentiation potential toward AV mesenchymal and valve endocardial cell (VEC) progenitor terminal states in endothelial-derived lineages. (k) Palantir results are robust when varying nearest-neighbor input, k. Plots show Pearson's correlation for pseudotime, differentiation (diff.) potential, and branch probability between pairs of Palantir runs. For box plots, the centre line represents the median value, where the bounds of the box span the interquartile range and whiskers represent minimum and maximum values, excluding outliers. Immunostaining results validated in $n \geq 3$ independent experiments. Scale bars, 100µm, 25µm in ROIs. AV, atrioventricular; AVC, atrioventricular canal; mes., mesenchyme; VECs, valve endocardial cells. Source Data are provided as a Source Data file.

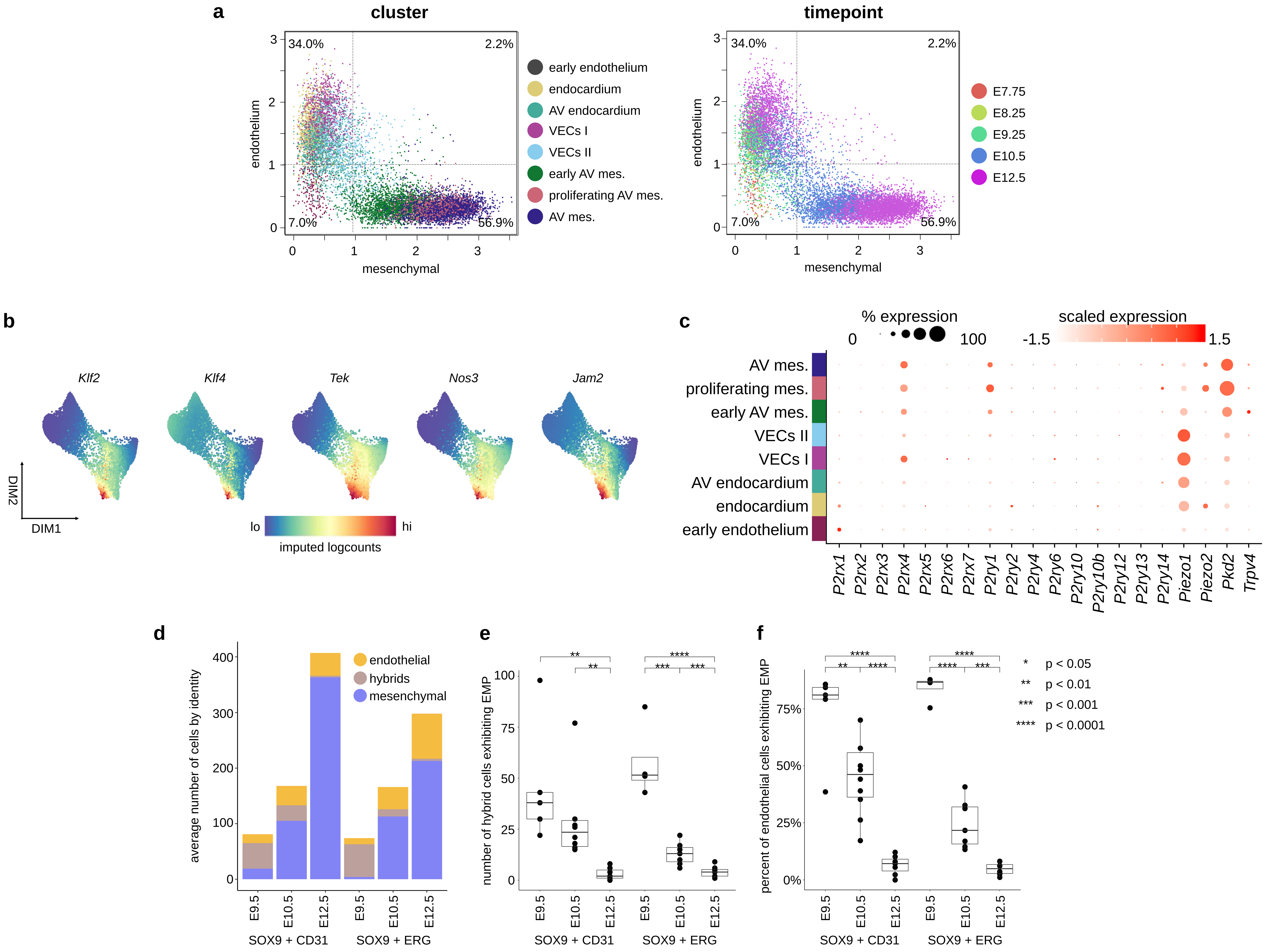

Supplementary Figure 3. Cell plasticity in endocardial-derived cell lineages during the endocardial-to-mesenchymal transition. Related to Fig. 3.

(a) Scatterplot comparing the endothelial and mesenchymal gene profiles either by timepoint or cluster. (b) Force-directed layouts displaying imputed gene expression of select fluid shear stress response genes. (c) Dot plot displaying expression of mechanoreceptors within endocardial-derived clusters during EndMT. Size of the dot represents proportion of the cluster that expresses each gene. Colour indicates level of expression. (d) Average number of endocardial, mesenchymal, and hybrid (EMP) cells in AVCs from E9.5, E10.5, and E12.5, showing notably reduced EMP by E12.5. (e) Number and (f) proportion of endothelial cells exhibiting EMP in AVCs from E9.5, E10.5, and E12.5, showing reduced numbers and overall proportions over time. All quantifications were performed on sections from n≥3 hearts (two-tailed unpaired t-test). For box plots, the line in the centre represents the median value, where the bounds of the box span the interquartile range and whiskers represent minimum and maximum values, excluding outliers. AV, atrioventricular; EMP, endothelial-mesenchymal plasticity; mes., mesenchyme; VECs, valve endocardial cells. Source Data are provided as a Source Data file.

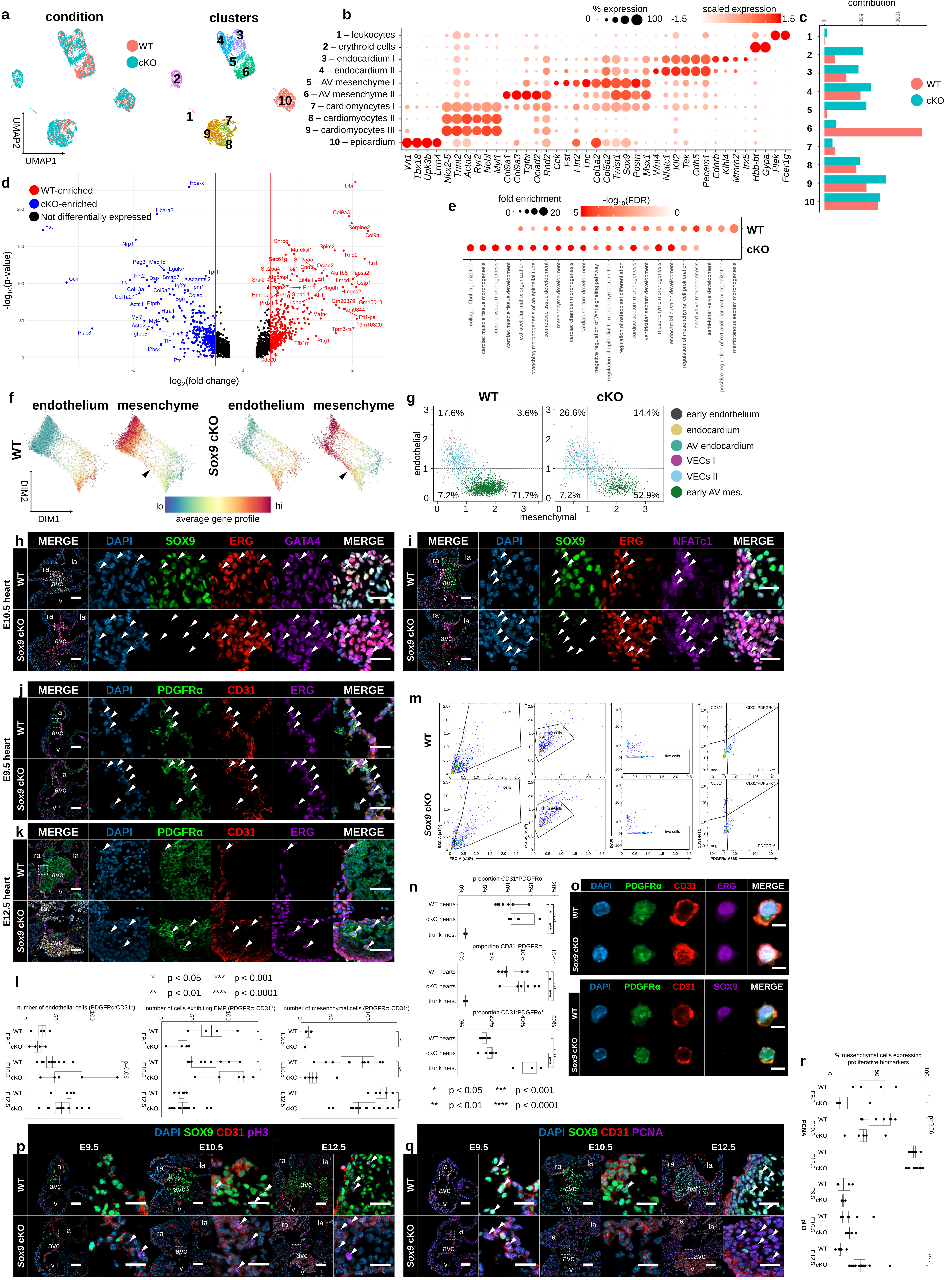

a
condition
clusters
WT
cKO
UMAP1
UMAP2
b
% expression
scaled expression
1 – leukocytes
2 – erythroid cells
3 – endocardium I
4 – endocardium II
5 – AV mesenchyme I
6 – AV mesenchyme II
7 – cardiomyocytes I
8 – cardiomyocytes II
9 – cardiomyocytes III
10 – epicardium
Wt1 Tbx18 Upk3b Lrrn4 Nkx2-5 Tnnt2 Acta2 Ryr2 Nebl Myl1 Col9a1 Col9a3 Tgfbi Ociad2 Rnd2 Cck Fst Flrt2 Tnc Col1a2 Col5a2 Twist1 Sox9 Postn Msx1 Wnt4 Nfatc1 Klf2 Tek Cdh5 Pecam1 Ednrb Klhl4 Mmrn2 Irx5 Hbb-bt Gypa Plek Fcer1g
c
contribution
d
WT-enriched
cKO-enriched
Not differentially expressed
log2(fold change)
-log10(p-value)
e
fold enrichment
-log10(FDR)
WT
cKO
collagen fibril organization
cardiac muscle tissue morphogenesis
muscle tissue morphogenesis
cardiac muscle tissue development
extracellular matrix organization
branching morphogenesis of an epithelial tube
connective tissue development
mesenchyme development
cardiac chamber morphogenesis
cardiac septum development
negative regulation of Wnt signaling pathway
regulation of epithelial to mesenchymal transition
regulation of osteoblast differentiation
cardiac septum morphogenesis
ventricular septum development
mesenchyme morphogenesis
endocardial cushion development
regulation of mesenchymal cell proliferation
heart valve morphogenesis
semi-lunar valve development
positive regulation of extracellular matrix organization
membranous septum morphogenesis
f
endothelium
mesenchyme
WT
Sox9 cKO
DIM1
DIM2
average gene profile
lo
hi
g
WT
cKO
endothelial
mesenchymal
early endothelium
endocardium
AV endocardium
VECs I
VECs II
early AV mes.
h
MERGE DAPI SOX9 ERG GATA4 MERGE
E10.5 heart
i
MERGE DAPI SOX9 ERG NFATc1 MERGE
j
MERGE DAPI PDGFRα CD31 ERG MERGE
E9.5 heart
k
MERGE DAPI PDGFRα CD31 ERG MERGE
E12.5 heart
l
* p < 0.05 *** p < 0.001
** p < 0.01 **** p < 0.0001
number of endothelial cells (PDGFRα-CD31+)
number of cells exhibiting EMP (PDGFRα+CD31+)
number of mesenchymal cells (PDGFRα+CD31-)
m
n
proportion CD31+PDGFRα-
proportion CD31+PDGFRα+
proportion CD31-PDGFRα+
WT hearts
cKO hearts
trunk mes.
o
DAPI PDGFRα CD31 ERG MERGE
DAPI PDGFRα CD31 SOX9 MERGE
p
DAPI SOX9 CD31 pH3
E9.5 E10.5 E12.5
q
DAPI SOX9 CD31 PCNA
E9.5 E10.5 E12.5
r
% mesenchymal cells expressing proliferative biomarkers
PCNA
pH3

Supplementary Figure 4. Comparison of WT and *Sox9* cKO atrioventricular single-cell libraries. Related to Fig. 4.

(a) UMAPs showing condition and cluster distribution of single-cell data generated from *Sox9* conditional knockout (cKO) and WT littermate atrioventricular (AV) canals. (b) Dot plot displaying select genes enriched within identified clusters. Size of the dot represents proportion of the cluster that expresses each gene. Colour indicates level of expression. Note that *Sox9* expression is retained in *Sox9* cKO mesenchyme due to capture of 5'-end upstream of flox site. (c) Contribution of WT and *Sox9* cKO single cells to each cluster identified at E10.5. (d) Volcano plots displaying differentially-expressed genes in AV mesenchyme in the WT and Sox9 cKO at E10.5. (e) Dot plot displaying shared and enriched gene ontology terms in WT and *Sox9* cKO mesenchymal clusters at E10.5. Size of the dot represents fold enrichment of the gene ontology term. Colour indicates significance based on false discovery rate (FDR). (f) Force-directed layouts displaying endothelial and mesenchymal gene profiles for endothelial-derived lineages from E10.5 WT and *Sox9* cKO libraries. Arrowheads indicate domains with increased mesenchymal gene profile expression in the *Sox9* cKO. (g) Scatterplot displaying endothelial and mesenchymal gene profiles for endothelial-derived lineages from E10.5 WT and *Sox9* cKO libraries, annotated by cluster. Immunostaining of AV canals at E10.5 for (h) SOX9, ERG, and GATA4 or (i) SOX9, ERG, and NFATc1 show increased distribution of GATA4+ and NFATc1+ endocardial, respectively (arrowheads). Immunostaining of AV canals for PDGFRα, CD31, and ERG at (j) E9.5 and (k) E12.5 reveal endocardial cells exhibiting mesenchymal features. (l) Quantifications of PDGFRα and CD31 immunostaining from Fig. 4f and Supplementary Fig. 4j-k, showing numbers of endocardial, mesenchymal, and hybrid (EMP) cells in WT and *Sox9* cKO AVCs from E9.5, E10.5, and E12.5 (two-tailed unpaired t-test). (m) Fluorescent-activated cell sorting (FACS) of WT and *Sox9* cKO whole, individual hearts with outflow tracts removed, showing (n) increased proportions of CD31+PDGFRα- endothelial cells and CD31 +PDGFRα+ within the *Sox9* cKO. E10.5 pharyngeal trunk mesenchyme (mes.) was used as positive PDGFRα control (two-tailed unpaired t-test). (o) Sorted CD31+PDGFRα+ single-cells show co-expression of endothelial and mesenchymal biomarkers. Note absence of SOX9 in a sorted CD31+PDGFRα+ single-cell from *Sox9* cKO. Immunostaining of proliferative markers, including (p) phospho-histone H3 (pH3) and (q) PCNA, along with markers of mesenchymal (SOX9) and endothelial (CD31) identity in WT and *Sox9* cKO AVCs from E9.5, E10.5, and E12.5. (r) Proportions of proliferating mesenchymal cells in WT and *Sox9* cKO AVCs at E9.5, E10.5, and E12.5 (two-tailed unpaired t-test). All quantifications were performed on sections from n≥3 hearts. For box plots, the centre line represents the median value, where the bounds of the box span the interquartile range and whiskers represent minimum and maximum values, excluding outliers. Immunostaining results validated in n ≥ 3 independent experiments. Scale bars, 100μm, 25μm in ROIs, 10μm for sorted single-cells. AV, atrioventricular; VECs, valve endocardial cells. Source Data are provided as a Source Data file.

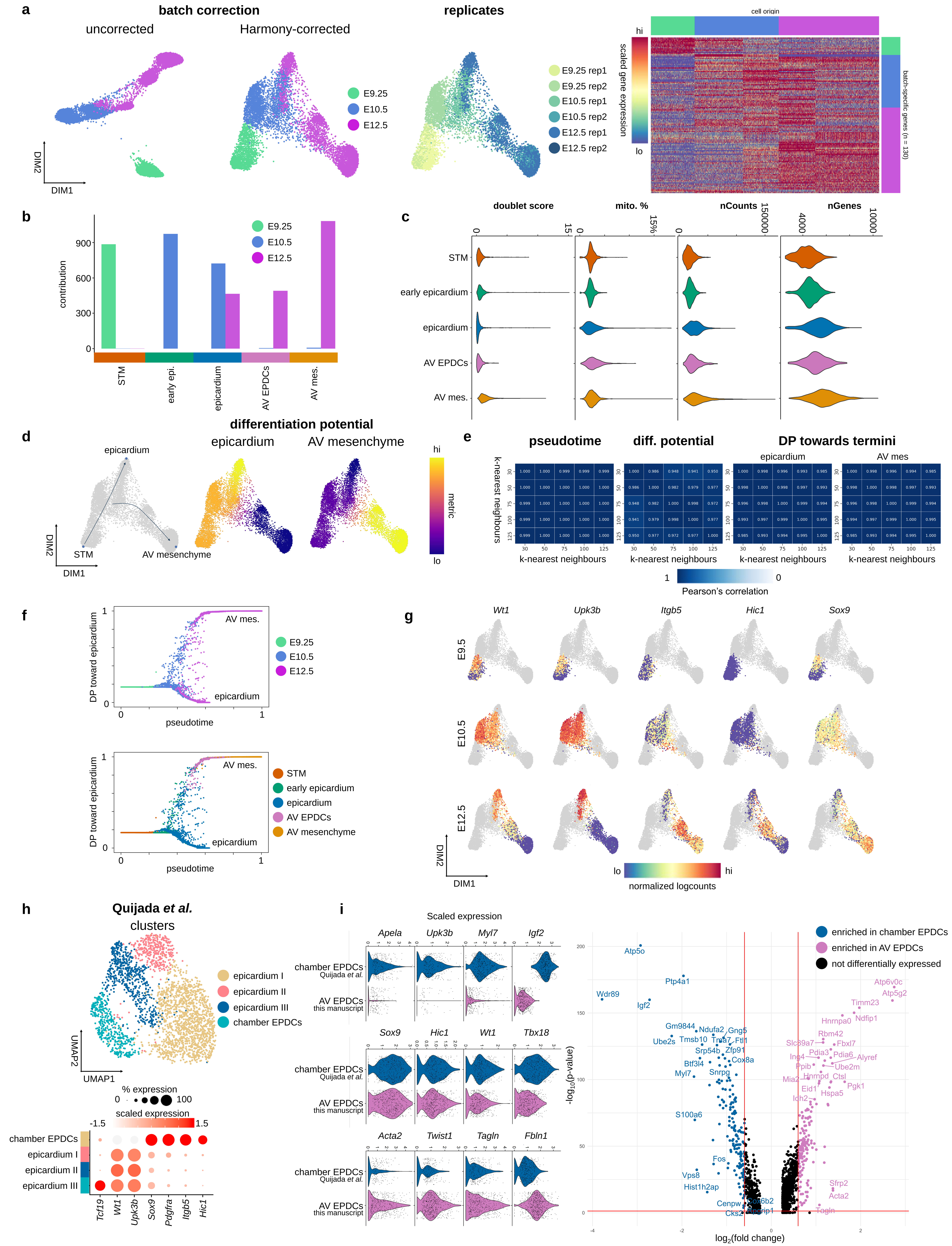
a
batch correction
uncorrected
Harmony-corrected
E9.25
E10.5
E12.5
DIM2
DIM1
replicates
E9.25 rep1
E9.25 rep2
E10.5 rep1
E10.5 rep2
E12.5 rep1
E12.5 rep2
cell origin
hi
lo
scaled gene expression
batch-specific genes (n = 130)
b
contribution
900
600
300
0
STM
early epi.
epicardium
AV EPDCs
AV mes.
c
doublet score
mito. %
nCounts
nGenes
STM
early epicardium
epicardium
AV EPDCs
AV mes.
d
differentiation potential
epicardium
AV mesenchyme
STM
hi
metric
lo
e
pseudotime
diff. potential
DP towards termini
epicardium
AV mes
k-nearest neighbours
Pearson's correlation
f
DP toward epicardium
pseudotime
AV mes.
epicardium
STM
early epicardium
epicardium
AV EPDCs
AV mesenchyme
g
Wt1
Upk3b
Itgb5
Hic1
Sox9
E9.5
E10.5
E12.5
lo
hi
normalized logcounts
h
Quijada et al.
clusters
epicardium I
epicardium II
epicardium III
chamber EPDCs
UMAP2
UMAP1
% expression
scaled expression
chamber EPDCs
epicardium I
epicardium II
epicardium III
Tcf19
Wt1
Upk3b
Sox9
Pdgfra
Itgb5
Hic1
i
Scaled expression
Apela
Upk3b
Myl7
Igf2
Sox9
Hic1
Wt1
Tbx18
Acta2
Twist1
Tagln
Fbln1
chamber EPDCs
Quijada et al.
AV EPDCs
this manuscript
enriched in chamber EPDCs
enriched in AV EPDCs
not differentially expressed
-log10(p-value)
log2(fold change)
Atp5o
Ptp4a1
Wdr89
Igf2
Atp6v0c
Atp5g2
Timm23
Ndfip1
Hnrnpa0
S100a6
Fos
Vps8
Hist1h2ap
Cenpw
Cks2
Sfrp2
Acta2
Tagln

Supplementary Figure 5. Characterization of epicardial diversity and differentiation during the epicardial-to-mesenchymal transition. Related to Fig. 5.

(a) Force-directed layouts and a heatmap showing timepoint distribution before and after Harmony correction as well as replicate distribution and batch-associated genes in epicardial lineages. (b) Timepoint contribution to each cluster identified in epicardial lineages. (c) Violin plots displaying doublet score, mitochondrial percent, nCounts, and nGenes for each cluster identified in epicardial lineages. (d) Force-directed layouts showing termini and rough trajectory for Palantir analysis and differentiation potential toward atrioventricular (AV) mesenchymal, and epicardial termini in epicardial lineages. (e) Palantir results are robust when varying nearest neighbor input, k. Plots show Pearson's correlation for pseudotime, differentiation (diff.) potential, and branch probability between pairs of Palantir runs. (f) Differentiation potential as a function of pseudotime for epicardial lineages progressing toward an epicardial terminus. Each dot represents a single-cell that is colour-coded by embryonic timepoint or cluster. (g) Expression of select epicardial and mesenchymal biomarkers, showing the precocious expression of *Itgb5* and *Hic1* by E10.5, suggesting early emergence of AV epicardial-derived cells (EPDCs). (h) Analysis of single-cell transcriptomic data of epicardial cells from Quijada *et al.* (2021) also reveals the presence of a distinct *Hic1*-expressing EPDC population. (i) Comparison of chamber EPDCs (from Quijada *et al.*) and AV EPDCs (from this manuscript) reveals transcriptional differences between these populations related to cell identity. epi., epicardium; mes., mesenchyme; STM, septum transversum mesenchyme. Source Data are provided as a Source Data file.

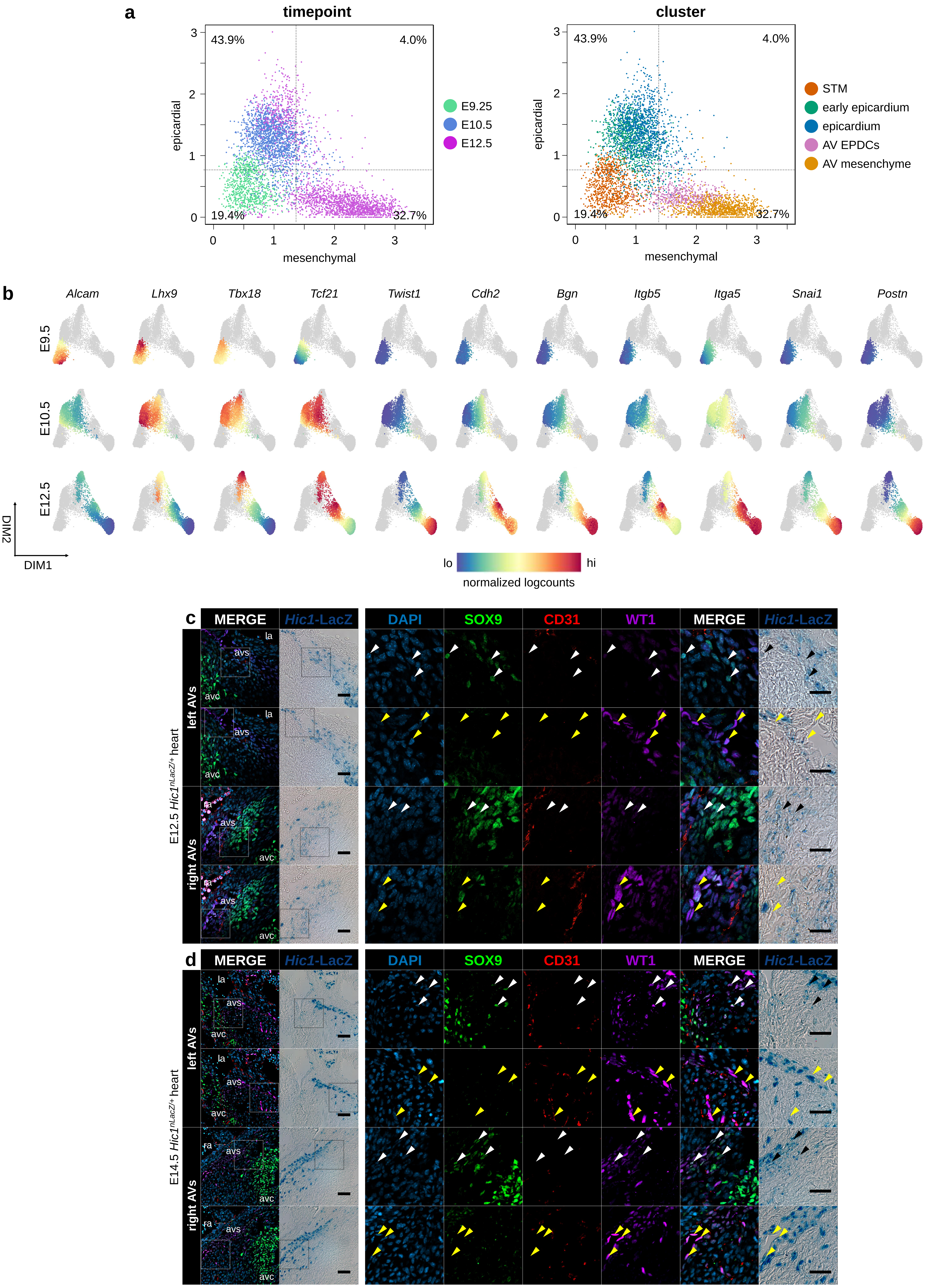

Supplementary Figure 6. Epicardial-to-mesenchymal plasticity during the epicardial-to-mesenchymal transition. Related to Fig. 6.

(a) Scatterplot comparing the epicardial and mesenchymal gene profiles either by timepoint or cluster. (b) Expression of select epicardial and mesenchymal biomarkers, showing shared expression of several mesenchymal factors between AV epicardial-derived cells (EPDCs) and mesenchymal cells at E12.5. SOX9, CD31, and WT1 staining on X-gal stained *Hic1*$^{nLacZ/+}$ hearts at (c) E12.5 and (d) E14.5. White and black arrowheads denote domains of low WT1 expression and high *Hic1*^{nLacZ}. Yellow arrowheads denote epithelial epicardial cells that express high WT1 and low *Hic1*^{nLacZ}. Immunostaining results validated in n ≥ 3 independent experiments. Scale bars, 100μm, 25μm in ROIs. epi., epicardium; mes., mesenchyme; STM, septum transversum mesenchyme.

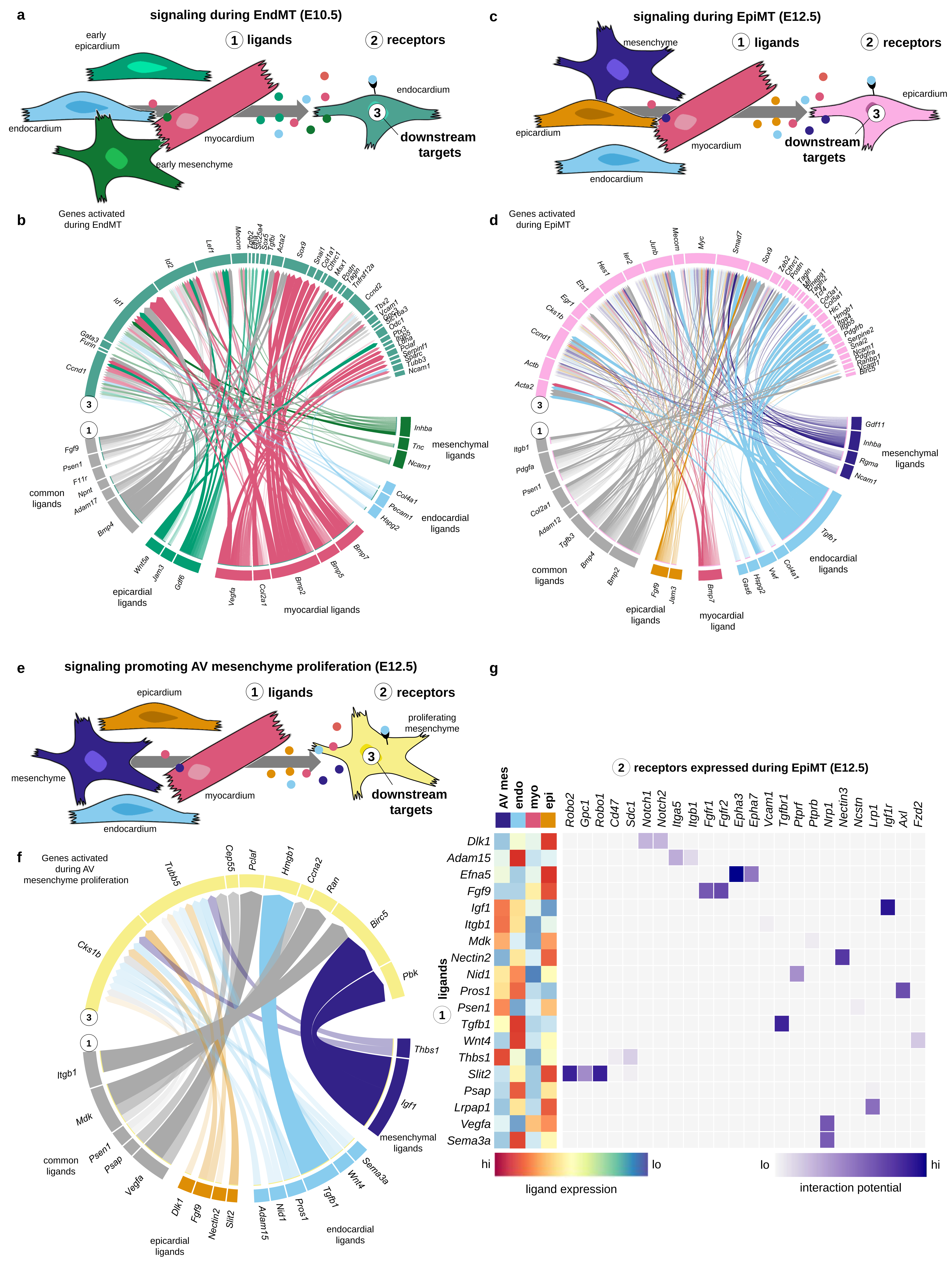

a
signaling during EndMT (E10.5)
1 ligands
2 receptors
early epicardium
endocardium
myocardium
early mesenchyme
endocardium
3
downstream targets
b
Genes activated during EndMT
Ccnd1
Gata3
Furin
Id1
Id2
Lef1
Mecom
Tgfb2
Sox9
Acta2
Snai1
Col1a1
Cthrc1
Msx1
Postn
Tagln
Tnfrsf12a
Ccnd2
Tbx2
Vcam1
Odc1
Ptx3
Itgb5
Pclaf
Serpinf1
Sparc
Tubb3
Ncam1
Inhba
Tnc
Ncam1
mesenchymal ligands
Col4a1
Pecam1
Hspg2
endocardial ligands
Bmp7
Bmp5
Bmp2
Col2a1
Vegfa
myocardial ligands
Gdf6
Jam3
Wnt5a
epicardial ligands
Bmp4
Adam17
Npnt
F11r
Psen1
Fgf9
common ligands
c
signaling during EpiMT (E12.5)
1 ligands
2 receptors
mesenchyme
epicardium
myocardium
endocardium
epicardium
3
downstream targets
d
Genes activated during EpiMT
Acta2
Actb
Ccnd1
Cks1b
Egr1
Ets1
Hes1
Ier2
Junb
Mecom
Myc
Smad7
Sox9
Zeb2
Cthrc1
Postn
Tagln
Tcf4
Col3a1
Col5a1
Hic1
Hmgb1
Itga4
Itgb5
Pdgfrb
Serpine2
Snai2
Ncam1
Pdgfra
Ranbp1
Vcam1
Birc5
Gdf11
Inhba
Rgma
Ncam1
mesenchymal ligands
Tgfb1
Col4a1
Vwf
Hspg2
Gas6
endocardial ligands
Bmp7
myocardial ligand
Jam3
Fgf9
epicardial ligands
Bmp2
Bmp4
Tgfb3
Adam12
Col2a1
Psen1
Pdgfa
Itgb1
common ligands
e
signaling promoting AV mesenchyme proliferation (E12.5)
1 ligands
2 receptors
epicardium
mesenchyme
myocardium
endocardium
proliferating mesenchyme
3
downstream targets
f
Genes activated during AV mesenchyme proliferation
Cks1b
Tubb5
Cep55
Pclaf
Hmgb1
Ccna2
Ran
Birc5
Pbk
Thbs1
Igf1
mesenchymal ligands
Sema3a
Wnt4
Tgfb1
Pros1
Nid1
Adam15
endocardial ligands
Slit2
Nectin2
Fgf9
Dlk1
epicardial ligands
Vegfa
Psap
Psen1
Mdk
Itgb1
common ligands
g
2 receptors expressed during EpiMT (E12.5)
AV mes
endo
myo
epi
Robo2
Gpc1
Robo1
Cd47
Sdc1
Notch1
Notch2
Itga5
Itgb1
Fgfr1
Fgfr2
Epha3
Epha7
Vcam1
Tgfbr1
Ptprf
Ptprb
Nrp1
Nectin3
Ncstn
Lrp1
Igf1r
Axl
Fzd2
1 ligands
Dlk1
Adam15
Efna5
Fgf9
Igf1
Itgb1
Mdk
Nectin2
Nid1
Pros1
Psen1
Tgfb1
Wnt4
Thbs1
Slit2
Psap
Lrpap1
Vegfa
Sema3a
hi
lo
ligand expression
lo
hi
interaction potential

Supplementary Figure 7. The cell signaling microenvironment of the atrioventricular canal. Related to Fig. 7.

(a) Schematic overview of EndMT signaling analysis, showing sender populations at E10.5 that may secrete (1) ligands to bind to (2) receptors expressed by endocardium, and stimulate (3) downstream target genes upregulated during EndMT. (b) Circle plots depicting links between (1) ligands and (3) downstream target genes upregulated during EndMT. (c) Schematic overview of EpiMT signaling analysis, showing sender populations at E12.5 that may secrete (1) ligands to bind to (2) receptors expressed by epicardium, and stimulate (3) downstream target genes upregulated during EpiMT. (d) Circle plots depicting links between (1) ligands and (3) downstream target genes upregulated during EpiMT. (e) Schematic overview of AV mesenchymal cell proliferation signaling analysis, showing sender populations at E12.5 that may secrete (1) ligands to bind to (2) receptors expressed by AV mesenchyme, and stimulate (3) downstream target genes upregulated in this proliferative state. (f) Circle plots depicting links between (1) ligands and (3) downstream target genes upregulated during AV mesenchymal cell proliferation. (g) A heatmap displaying select ligand-receptor interactions signaling toward AV mesenchymal cells at E12.5 that may promote cell proliferation, including scaled and log-normalized expression of ligands from sender populations. For circle plots, link colours indicate ligand sender populations, whereas width and opacity of links correlate to ligand-receptor interaction weights and ligand activity scores, respectively. AV, atrioventricular; endo, endocardium; epi, epicardium; myo, myocardium.
