# Supplementary material for: Cell diversity and plasticity during atrioventricular heart valve EMTs
Source: Nat Commun. 2023 Sep 9;14:5567. doi: 10.1038/s41467-023-41279-6 (PMC10492828; doi:10.1038/s41467-023-41279-6)
Supplement: Supplementary file 3 — Description of Additional Supplementary Files [file 41467_2023_41279_MOESM3_ESM.pdf]

Description of Additional Supplementary Files

File Name: **Supplementary Data 1 – Quality Control**
Description: Summary of quality metrics for single-cell libraries analyzed.

File Name: **Supplementary Data 2 – Differential Gene Analysis complete**
Description: Differentially-expressed genes (two-sided unpaired Wilcoxon rank sum test) for the complete dataset, EndMT and EpiMT subsets, and the *Sox9* cKO data.

File Name: **Supplementary Data 3 - Batch Effect Genes**
Description: Batch-associated genes (two-sided unpaired Wilcoxon rank sum test) and gene ontology analysis.

File Name: **Supplementary Data 4 - Gene lists for profile analysis**
Description: Genes used for endothelial, mesenchymal, epicardial, and fluid shear stress gene profile analysis.

File Name: **Supplementary Data 5 - Cell signaling analysis supplementary tables**
Description: Complete NicheNet data.

File Name: **Supplementary Data 6 - Reagents and Resources**
Description: Detailed list of reagents and resources, including antibody information, used in this manuscript.

File Name: **Supplementary Data 7 - Genotyping primers**
Description: List of primers used for genotyping.

File Name: **Supplementary Data 8 - Gene lists for NicheNet analysis**
Description: Target genes of interest for NicheNet analysis.
