# Supplementary material for: Cell diversity and plasticity during atrioventricular heart valve EMTs
Source: Nat Commun. 2023 Sep 9;14:5567. doi: 10.1038/s41467-023-41279-6 (PMC10492828; doi:10.1038/s41467-023-41279-6)
Supplement: Supplementary file 12 — Reporting Summary [file 41467_2023_41279_MOESM12_ESM.pdf]

nature portfolio

Corresponding author(s): Pamela A. Hoodless

Last updated by author(s): Aug 17, 2023

Reporting Summary

Nature Portfolio wishes to improve the reproducibility of the work that we publish. This form provides structure for consistency and transparency in reporting. For further information on Nature Portfolio policies, see our Editorial Policies and the Editorial Policy Checklist.

Statistics

For all statistical analyses, confirm that the following items are present in the figure legend, table legend, main text, or Methods section.

n/a	Confirmed	
☐	☒	The exact sample size (*n*) for each experimental group/condition, given as a discrete number and unit of measurement
☐	☒	A statement on whether measurements were taken from distinct samples or whether the same sample was measured repeatedly
☐	☒	The statistical test(s) used AND whether they are one- or two-sided<br>*Only common tests should be described solely by name; describe more complex techniques in the Methods section.*
☒	☐	A description of all covariates tested
☐	☒	A description of any assumptions or corrections, such as tests of normality and adjustment for multiple comparisons
☐	☒	A full description of the statistical parameters including central tendency (e.g. means) or other basic estimates (e.g. regression coefficient) AND variation (e.g. standard deviation) or associated estimates of uncertainty (e.g. confidence intervals)
☐	☒	For null hypothesis testing, the test statistic (e.g. *F*, *t*, *r*) with confidence intervals, effect sizes, degrees of freedom and *P* value noted<br>*Give P values as exact values whenever suitable.*
☒	☐	For Bayesian analysis, information on the choice of priors and Markov chain Monte Carlo settings
☒	☐	For hierarchical and complex designs, identification of the appropriate level for tests and full reporting of outcomes
☐	☒	Estimates of effect sizes (e.g. Cohen's *d*, Pearson's *r*), indicating how they were calculated

Our web collection on statistics for biologists contains articles on many of the points above.

Software and code

Policy information about availability of computer code

Data collection	Images were captured with NIS Elements (https://www.microscope.healthcare.nikon.com/products/software/nis-elements), single-cell transcriptomes were sequenced on a NextSeq 2000.
Data analysis	ImageJ v1.53k, Cellranger v5.0, R v4.0.3, Seurat v4.0.4, NicheNet v1.0.0, circlize v0.4.13, scran v1.18.7, scater v1.18.6, dplyr v1.0.7, batchelor v1.6.3, Python v3.7, Harmony v0.1.4, Palantir v1.0.0, pandas v1.4.2, numpy v1.22.3, scipy v1.8.0, matplotlib v3.5.1, seaborn v0.11.2, rpy2 v3.0.5

For manuscripts utilizing custom algorithms or software that are central to the research but not yet described in published literature, software must be made available to editors and reviewers. We strongly encourage code deposition in a community repository (e.g. GitHub). See the Nature Portfolio guidelines for submitting code & software for further information.

Data

Policy information about availability of data

All manuscripts must include a data availability statement. This statement should provide the following information, where applicable:

- Accession codes, unique identifiers, or web links for publicly available datasets
- A description of any restrictions on data availability
- For clinical datasets or third party data, please ensure that the statement adheres to our policy

All single-cell RNA-sequencing data generated during this study are deposited in the ArrayExpress database at EMBL-EBI (www.ebi.ac.uk/arrayexpress) under the accession number E-MTAB-11795 (https://www.ebi.ac.uk/biostudies/arrayexpress/studies/E-MTAB-11795?query=E-MTAB-11795). The processed data are also deposited on the Broad Single0cell Portal under the accession number SCP1897 (https://singlecell.broadinstitute.org/single_cell/study/SCP1897/single-cell-atlas-of-embryonic-heart-valve-emts)

Field-specific reporting

Please select the one below that is the best fit for your research. If you are not sure, read the appropriate sections before making your selection.

☒ Life sciences ☐ Behavioural & social sciences ☐ Ecological, evolutionary & environmental sciences

For a reference copy of the document with all sections, see nature.com/documents/nr-reporting-summary-flat.pdf

Life sciences study design

All studies must disclose on these points even when the disclosure is negative.

Sample size	Sample sizes were not statistically determined, but instead to maximize the number of recovered cells from each experiment.
Data exclusions	No exclusion was applied.
Replication	Single-cell RNA-seq samples from wildtype atrioventricular canals were generated in duplicate. A single replicate of the Sox9 Tie2-cre cKO atrioventricular canal library was generated.
Randomization	No randomization was performed.
Blinding	No blinding was done for these analyses.

Reporting for specific materials, systems and methods

We require information from authors about some types of materials, experimental systems and methods used in many studies. Here, indicate whether each material, system or method listed is relevant to your study. If you are not sure if a list item applies to your research, read the appropriate section before selecting a response.

Materials & experimental systems

n/a	Involved in the study	
☐	☒	Antibodies
☒	☐	Eukaryotic cell lines
☒	☐	Palaeontology and archaeology
☐	☒	Animals and other organisms
☒	☐	Human research participants
☒	☐	Clinical data
☒	☐	Dual use research of concern

Methods

n/a	Involved in the study	
☒	☐	ChIP-seq
☐	☒	Flow cytometry
☒	☐	MRI-based neuroimaging

Antibodies

Antibodies used	Rat anti-CD31 (1:50 Biolegend, Cat # 105205), Rabbit anti-ERG (1:50 Abcam, Cat # ab92513), Mouse anti-GATA4 (1:50 Santa Cruz, Cat # sc-25310), Rabbit anti-MCM-2 (1:100 Abcam, Cat # ab108935), Mouse anti-NFATc1 (1:15 DSHB, Cat # 7A6-s), Rabbit anti-PCNA (1:100 Abcam, Cat # ab70472), Goat anti-PDGFRα (1:50 R&D, Cat # AF1062), Rabbit anti-pHH3 (1:100 Upstate, Cat # 06-570), Goat anti-SOX9 (1:50 R&D, Cat # AF3075), Rabbit anti-SOX9 (1:500 Millipore, Cat # ab5535), Mouse anti-TNNT2 (1:15 DSHB, CAT # RV-C2-s), Rabbit anti-VIM (1:50 Cell Signaling, Cat # 5741S), Rabbit anti-WT1 (1:50 Abcam, Cat # ab89901), Rat anti-Ter119, APC (1:100 Thermo, Cat # 17-5921-82), Donkey anti-goat A488 (1:500 Thermo, Cat # A11055), Goat anti-rabbit A488 (1:500 Thermo, Cat # A11008), Donkey anti-goat A568 (1:500 Thermo, Cat # A11057), Donkey anti-mouse A568 (1:500 Thermo, Cat # A10037), Donkey anti-rabbit A568 (1:500 Thermo, Cat # A10042), Donkey anti-rat A594 (1:500 Thermo, Cat # A21209), Donkey anti-mouse A647 (1:500 Thermo, Cat # A31571), Donkey anti-rabbit A647 (1:500 Thermo, Cat # A31573)
Validation	Antibodies were validated on whole wildtype mouse embryo cryosections. Secondary antibodies were validated on tissue cryosections without primary antibodies.

Animals and other organisms

Policy information about studies involving animals; ARRIVE guidelines recommended for reporting animal research

Laboratory animals	All mice were maintained in accordance with the guidelines of the University of British Columbia' Animal Care Committee's Standards under protocol numbers A20-0281, A20-0282, and A19-0221 under pathogen-free conditions. Mice were housed in a temperature-controlled (18-23C, 40–60% humidity) and enriched environment, with a 12h light/dark cycle, and provided standard chow and water. E10.5 rep1 - CD-1, E10.5 rep2 - Tie2-cre, E10.5 Sox9 cKO - Tie2-cre;Sox9fl/fl, E12.5 - Tie2-cre;tdTomato (Ai9), Hic1-LacZ histology - Hic1-nLacZ/+. Embryo sex was not determined as sex-specific cues are not thought to play a significant role at the developmental timepoints assayed.

Wild animals | This study did not involve wild animals.

Field-collected samples | This study did not collect samples from the field.

Ethics oversight | All mice were maintained in accordance with the University of British Columbia's Animal Care Committee's standards under protocol numbers A20-0281, A20-0282, and A19-0221 in pathogen-free conditions.

Note that full information on the approval of the study protocol must also be provided in the manuscript.

Flow Cytometry

Plots

Confirm that:

☒ The axis labels state the marker and fluorochrome used (e.g. CD4-FITC).

☒ The axis scales are clearly visible. Include numbers along axes only for bottom left plot of group (a 'group' is an analysis of identical markers).

☒ All plots are contour plots with outliers or pseudocolor plots.

☒ A numerical value for number of cells or percentage (with statistics) is provided.

Methodology

Sample preparation | See methods for detailed descriptions. For E10.5 rep1, AVCs were collected using fine dissecting forceps, ensuring the removal of the outflow tract, atria, and ventricles. The AVC, including AV cushions as well as overlying myocardium and epicardium were retained. AVCs were washed in PBS and incubated for five minutes at 37°C in 1ml PDEF (10 µg/ml DNAse I, 4mM EDTA, and 2% fetal bovine serum [FBS] in PBS) with dispase/collagenase (2.5µU/ml dispase, 0.0025% collagenase type I) to dissociate tissues into single-cells. Tissues were gently triturated using a 1000µl pipette before and after enzymatic digestion to facilitate dissociation. Single-cell suspensions were filtered through 40µm cell strainers into 5ml FACS tubes to remove debris and incompletely dissociated cells. The strainers were washed with PDEF to collect the remaining cells. The filtrate was subsequently centrifuged at 1000g for four minutes to sediment single cells and the supernatant was removed. For all other libraries, tissues were collected from E10.5 and E12.5 embryonic hearts, ensuring removal of cardiac chambers and outflow tract. AVCs were washed for 5 minutes in PBS to remove red blood cells. PBS was removed and replaced with 250-1000µl TrypLE Express. Tissues were gently triturated, then incubated at 37°C for 5-15 minutes until the suspension was homogeneous and pieces of tissue were not visible. Cell suspensions were gently triturated part way through for longer incubations and again at the end. Single-cell suspensions were filtered through 40µm cell strainers into 5ml FACS tubes to remove debris and incompletely dissociated cells. The strainers were washed with PDEF to collect the remaining cells. The filtrate was subsequently centrifuged at 1000g for four minutes to sediment single cells and the supernatant was removed. For E10.5 Sox9 cKO and littermate control libraries, AVCs were collected and left on ice for 90 minutes while genotyping was performed. Sox9fl/fl;Tie2-cre cKO AVCs or Sox9+/+ and Sox9fl/+;Tie2-cre AVCs were then pooled, dissociated, and re-suspended in PDEF before preparing single-cell libraries. At E12.5, cells were re-suspended in PDEF and stained with 1:5000 300um DAPI. To remove hematopoietic populations, dissociated AVC suspensions were resuspended in 2% FBS in PBS, and stained with APC-conjugated anti-Ter119 (1:200) at 4°C for 15 minutes. After staining, cell suspensions were washed three times in PDEF, centrifuging at 1000g for four minutes, and were ultimately resuspended in PDEF.

Instrument | Sorting was done using a FACSARIA III cell sorter (BD Biosciences) with a 130µm nozzle in purity mode.

Software | FACS DIVA

Cell population abundance | From each tissue, we sorted several thousand cells

Gating strategy | SSC vs FSC, a clear population of cell emerged, and a gate was drawn to exclude small debris. SSC-H vs. SSC-W excluded cell aggregated. Samples were sorted to exclude dead (DAPI+) cells, which were less than 5% of a given suspension, and at E12.5, Ter119+ erythrocytes were excluded.

☒ Tick this box to confirm that a figure exemplifying the gating strategy is provided in the Supplementary Information.
